# Supplementary material for: Synthetic data in health care: A narrative review
Source: PLOS Digit Health. 2023 Jan 6;2(1):e0000082. doi: 10.1371/journal.pdig.0000082 (PMC9931305; doi:10.1371/journal.pdig.0000082)
Supplement: Attachment — Submitted filename: Response to Reviewers.docx [file pdig.0000082.s001.docx]

To the reviewers and editors,

Greetings! We appreciate the feedback and the opportunity to respond to the valuable comments raised for our manuscript entitled “Synthetic data in health care: A narrative review” (PDIG-D-22-00188). The table below summarizes our response to each of the comments.

| **Comments** | **Responses** |
| --- | --- |
| PLOS Digital Health Publication Criteria: |  |
| I will note that the manuscript does not adhere to the Submission Guidelines "Manuscript Organization" for this journal. For example, there is no Materials and Methods section. A common practice for the methodology section of a review paper is to describe use of "PubMed, Scopus, etc." including search terms, how many articles the authors found, how they filtered or down-selected relevant items, counts, etc. | Methods section added. |
| The phrase "While real or the original data" is awkward English. Possibly "While the original real data..." | Changed as suggested. |
| The phrase "synthetic dataset contains all or a subset of not real (or fake) microdata" is awkward English. Suggest rephrasing or adding commas. For example, "synthetic datasets consist entirely of, or contain a subset of, not real (or fake) microdata". | Changed as suggested. |
| The sentence "More about formal definition and types in the section" is not complete. | This was meant to refer to the next main section which is the results section. I have changed it to: “The formal definition and types are further discussed in the succeeding section.” |
| General Comments: Reviewer 1 |  |
| Overall, I think the article provides a good overview of the use of synthetic data in health care, including some trade-offs, and it is generally well-written. Several niche topics I thought would be missed were included, so there is some depth as well. | Thanks! |
| Consider explicitly naming or citing notable government regulations such as HIPAA or the GDPR. It is not essential, but it seems strange not to, and it could lead the interested reader to find out more. | Mentioned HIPAA in the introduction section |
| I suggest changing "When an attacker has access to the synthetic data and the model code used to generate it" to "When an attacker has access to the synthetic data and the generative model used to create that data" as an explicit clarification, because this risk is systemic to generative adversarial models in particular and you don't need the "code" (which I interpret as "source code") you just need the resulting model. | Thank you very much for this – I have updated the statement based on this. |
| While GANs are addressed broadly, it might be worth mentioning that these techniques are currently most useful for generating synthetic imagery (e.g., ultrasounds, MRIs). While some of the other techniques and data focus on structured longitudinal data (e.g., the CMS desynpuf data). The point is, there is a lot of variety in what synthetic health data is. | Mentioned models in the generating synthetic images in the software and tools section. |
| The "SyntheticUSA" project appears to be inactive for 5 years. It might be better to reference more recent work from the Synthea group. For example, the "coherent data set" (https://doi.org/10.3390/electronics11081199) or the "FDA/VHA COVID-19 challenge"  https://doi.org/10.1016/j.ibmed.2020.100007  https://precision.fda.gov/challenges/11  https://precision.fda.gov/challenges/11/results | SyntheticUSA was removed and information about the “Coherent dataset” was added in the examples section |
| Validation is an important topic that is covered at the end of the Discussion section. For many synthetic data sets, and some of the tools used to generate those data sets, validation is self-reported. Questions for the authors to consider discussing: How does a 3rd party validate or evaluate fit-for-purpose of synthetic data if they don't have access to the original real data nor the model used to generate that data? What level of transparency or assurance is required by different use-cases? | This is an area that we think is yet to be explored and we hope that the paper could start that discussion. As mentioned in the paper, according to existing literature, there is no benchmark in validating synthetic data, but frameworks have been proposed. |
| Final thought for author consideration: recent hot topics not covered in this review include "digital twins" and "synthetic" or "in silico" clinical trials. "Digital twin" is an umbrella term that includes many things (including modeling and simulation, big data, etc), sometimes to include synthetic health data. There is also recent work on "in silico" clinical trials using a variety of techniques, including models specifically built for that purpose and synthetic health data. Consider discussing these uses. | The in silico clinical trial was included under the “Simulation studies and predictive analytics” subsection |
| General Comments: Reviewer 2 |  |
| + The authors need to explain what is the delta compared to this paper that was published recently (or at least cite it and provide incremental information):  https://link.springer.com/article/10.1007/s44163-021-00016-y | I believe that the two papers complement each other. The paper mentioned focuses on the general use cases of synthetic data. On the other hand, our paper highlights how synthetic data could be used in healthcare. While there are a few that overlaps – our paper hopes to serve as a primer to the topic, targeting those in healthcare by providing specific examples of how it could be used for their healthcare use cases. |
| + There are more vendors in this space beyond MDClone, such as Syntegra, Replica Analytics, mostly.ai, hazy, Diveplane, and more. The discussion of commercial tools is not even illustrative. | In the paper, we mentioned those that were used in the papers. When we did a quick PubMed search of the tools mentioned here an no result for the majority. A statement that this is not an exhaustive list was added to signal the readers that there are other tools aside from what is listed in the paper. We also changed the heading of this to “Examples of software and tools in generating synthetic data” |
| + There are many more open source tools that are available for generating synthetic data, such as the multiple variants of GANs that have been developed for health data. | Again, we recognize that there are other tools and what we provided in the paper are some examples. Exploring how synthetic data is generated could be a paper on its own. We focus on the use case side. |
| + There are more publicly available synthetic health datasets from France, the UK, the Netherlands, and Korea. These demonstrate the diversity of synthetic data and the global adoption of the approach. | In the paper, we highlight examples of datasets that are designed for public use. In the statement, we also recognized that there are other, mostly project-specific, datasets. The examples provided here illustrate the different utility of synthetic data as well as something that researchers could use as a starting point when using synthetic data. If the editors see the necessity to really add more examples from other countries, we can work on another section (pending the availability of information re its utility). |
| + While it is tempting to draw general conclusions, there are a few things to note: (a) using references from the early 2000's to comment on privacy and utility risks ignores the significant work that has been in these areas over the last few years, and (b) the utility and privacy risks from synthetic data will depend on the generative models that are used and there is quite a bit of heterogeneity - a deeper analysis of these variations would give a more meaningful set of conclusions. | For A:  Since the comment was not specific to a section, I did look into the references related to privacy and I think there was one in 2003. Since we mentioned the year in the definition section, I also assumed that it could be it. As a response, the section where the year was mentioned and the one with privacy reference in 2003 provide different perspectives on how synthetic data was defined. As mentioned in the paper, the majority of the paper on synthetic data would refer to definitions cited/enhanced in the 2000’s. We also mentioned newer definitions in contrast to the old ones. Please let us know if there are specific references that we would need to address.  For B:  We currently can’t find resources that would help us think about how the variations in models could impact utility and privacy. However, in the paper, we presented how the composition of the synthetic data or how synthetic the data is data can influence its utility. Again, this is an emerging topic and most literature cited in the discussion section presented also the risks in general. |
| While I appreciate that this is only a narrative review, the gap from a good coverage of the topic is quite large that I do not think it gives a reasonable representation of the topic at all. | We recognize that synthetic data is an emerging topic, and we tried our best to summarize what is known to inform healthcare workers and researchers who are exploring synthetic data. The paper also focused on the use cases and examples to illustrate and encourage more research in the area. Considering our target audience, those who are trying to learn about this topic, we kept the depth in a way that a non-technical audience could get it. We hope that after priming their interest through the paper, they will continue to explore the area following their specific use cases and be guided by additional resources that would expand the technicalities of synthetic data. |

Sincerely,

**Aldren Gonzales**

Lead/Corresponding Author
