# Supplementary material for: Synthetic data in health care: A narrative review
Source: PLOS Digit Health. 2023 Jan 6;2(1):e0000082. doi: 10.1371/journal.pdig.0000082 (PMC9931305; doi:10.1371/journal.pdig.0000082)
Supplement: Attachment — Submitted filename: Response to Reviewers_12-2.docx [file pdig.0000082.s002.docx]

Thanks again for the critical inputs and the opportunity to revise our manuscript:

| **Comment** | **Action** |
| --- | --- |
| Reviewer #1: All of my comments were adequately addressed. | No action |
| Reviewer #3: The paper offers a review and discussion regarding synthetic data. As the paper attempts to be comprehensive, it lacks in depth assessments of validation. To contribute to the significance of the paper, I find it particularly important to include more regard to validation, and recommend to add a section that will review validation works.  In particular, the authors have missed an essential validation study published recently (2020), which is relevant in general for validation of synthetic data for use for medical research, and in particular concerning the MDClone system (see reference below). The paper performs a comparative validation study based on 5 research questions and datasets, and concludes important pros and cons, with no commercial bias. The paper also discusses available alternatives and approaches for generation of synthetic data.  Please cite the paper (i) when referring MDCLone and (ii) when discussing validation. In addition, emphasize the added value of the proposed manuscript in relation to the review conducted in the introduction in that paper.  "Analyzing Medical Research Results Based on Synthetic Data and Their Relation to Real Data Results: Systematic Comparison From Five Observational Studies." Reiner Benaim et al, JMIR (2020): DOI: 10.2196/16492, PMID: 32130148, PMCID: PMC7059086. | We have cited the paper in the MDClone section. We have also expanded the validation section in the discussion – including illustrating examples of validation approaches including the paper mentioned here. |
| References list | We have reviewed the reference list and updated those with missing information. As of writing, no cited papers have been retracted |
